# Effect of Coffee Consumption on Postoperative Ileus after Colorectal Surgery: A Meta-Analysis of Randomized Controlled Trials

**DOI:** 10.1155/2022/8029600

**Published:** 2022-06-08

**Authors:** Junjia Zhu, Wenlong Xu, Qi Sun, Jun Geng, Yifeng Yu, Zhenguo Zhao

**Affiliations:** ^1^Department of General Surgery, The Affiliated Jiangyin Hospital of Southeast University Medical College, Jiangyin 214400, China; ^2^Department of Anesthesiology, The Affiliated Jiangyin Hospital of Southeast University Medical College, Jiangyin 214400, China

## Abstract

**Background:**

Postoperative ileus (POI) is an important complication after elective colorectal surgery, which prolongs hospital stay and increases hospital costs. Coffee has been reported to be beneficial for the recovery of gastrointestinal function. We aimed to investigate the effectiveness of coffee consumption in the treatment of POI, following elective colorectal surgery.

**Methods:**

A comprehensive literature search for medical subject heading (MeSH) terms, including coffee, caffeine, colon, rectum, and colorectal surgery was conducted in PubMed, Embase, and Cochrane Library until November 2021. A meta-analysis of postoperative outcomes was conducted to assess the effectiveness of coffee consumption on POI after colorectal surgery.

**Results:**

726 articles were identified and six RCTs that captured 416 patients were included. The time to first defecation was reduced with postoperative coffee consumption compared to the control group (mean difference = −15.03 h; 95% confidence interval: -17.79, -12.26; *P* < 0.00001). There was no difference in time to first flatus, time to tolerance for solid food, length of hospital stay, use of laxatives, reinsertion of nasogastric tube, need for reoperation, postoperative complications, and anastomotic leak between the groups. Coffee did not have any adverse effects.

**Conclusion:**

The current literature revealed that postoperative coffee consumption shortened the time to first defecation following elective colorectal surgery. Large sample and tightly controlled multicenter randomized clinical trials are needed to offer a more accurate evaluation of the efficacy of coffee.

## 1. Introduction

Postoperative ileus (POI) is an important cause of extended hospitalization, following abdominal surgery, especially after a colon surgery [1, 2]. The incidence of POI ranges from 10 to 20% and lasts from 3 to 5 days after colorectal resection [3, 4]. Common symptoms associated with ileus include anorexia, nausea, vomiting, intestinal cramps, abdominal discomfort, and lack of flatus or passage of stool [1]. Recent studies have identified the main mechanisms of POI, including neurogenic dysfunction, use of analgesics (such as opioids), intestinal inflammation, and surgical procedures [5–7]. Prolonged hospital stay and complications caused by POI have a significant impact on healthcare services [8], and it has been found to increase hospital spending by 750 million USD annually in the USA [1].

Treatment of postoperative intestinal obstruction includes nasogastric tube decompression, correction of electrolyte disturbances, and analgesia [9]. Considering its serious effects and the lack of effective therapies, many enhanced recovery protocols have been proposed to shorten POI [10]. However, not all measures offer a complete success [10].

Coffee is a widely consumed beverage with well-known effects on the central nervous system and cardiovascular system [11]. It is associated with an increase in bowel function in healthy individuals [12]. Recently, several small randomized clinical trials reported positive clinical outcomes of coffee use in the management of POI; however, the results are not completely consistent [13, 14]. Güngördük et al. concluded that the administration of coffee reduces POI in a meta-analysis of four trials [15]. However, the four trials in this analysis covered both gynecological and colorectal surgeries.

We performed this systematic review and meta-analysis to investigate the efficacy of coffee consumption on POI after elective colorectal surgery.

## 2. Materials and Methods

### 2.1. Identification of Trials

We conducted the literature search according to the Preferred Reporting Items for Systematic Reviews and Meta-analyses (PRISMA) guidelines [16], in order to build an evidence base for the assessment of postoperative outcomes of colorectal surgery with or without the administration of coffee and caffeine. We performed the comprehensive literature search through PubMed, Embase, and Cochrane Library until November 2021, using medical subject heading (MeSH) terms: coffee, caffeine, colon, rectum, and colorectal surgery.

### 2.2. Selection Criteria

Two reviewers screened the titles for relevance before assessing the abstracts and full-text articles. Disagreements were resolved by discussing with a third reviewer. The following PICO criteria were created to conduct an appropriate screen for literatures: In adults who undergo colorectal surgery (P), does the administration of coffee (I), compared to a comparative control group (C), decreases the recovery time of bowel function after the operation (O)? There were no language or time restrictions. For subsequent analyses, only randomized controlled trials (RCTs) were included. Abstracts from conferences, editorial reviews, letters, and nonhuman studies were not included in the study. Most recent publications were selected if the papers discussed the same research population.

### 2.3. Data Extraction

Data were extracted and checked by two reviewers. Differences in judgments were resolved by discussing with a third reviewer. Information on authors' names, publication year, study country and size, patient demographics, surgical information, and intervention information were extracted from each study.

The primary outcome of POI was defined as the time to first defecation. Secondary outcomes included time to first flatus, time to tolerance for solid food, length of hospital stay (LOS), use of any laxatives, need for reoperation, reinsertion of nasogastric tube, postoperative complications, and anastomotic leak.

### 2.4. Quality Assessment

Two reviewers assessed the risk of study bias using the Cochrane tool for assessing risk of bias independently [17]. Studies were assessed as high, low, or unclear risk of bias using seven items, including random sequence generation, allocation concealment, blinding of participants and personnel, blinding of outcome assessment, incomplete outcome data, selective reporting, and other bias. Discrepancies were resolved by discussing with a third reviewer.

### 2.5. Data Analysis

A meta-analysis was carried out with Review Manager (version 5.3). The risk ratio (RR) and 95% confidence interval (CI) were calculated for dichotomous variables using the Mantel–Haenszel method [18]. The mean difference (MD) and 95% CI were estimated using the inverse variance method for continuous variables [19]. If the median, interquartile range (IQR), or range were available, the mean and standard deviation (SD) were calculated via the Box-Cox method [20].

Statistical heterogeneity was evaluated using the *I*^2^ statistic and *P* value of the *I*^2^ test. Interstudy statistical heterogeneity was regarded as nonsignificant when *I*^2^ > 50% and *P* < 0.1, in which case, a random-effect model was applied. Otherwise, a fixed-effect model was used.

Publication bias was not performed because of the small number of included studies. A *P* value < 0.05 was considered significant.

We performed sensitivity analysis by repeating the meta-analysis after excluding one study at a time to explore potential sources of heterogeneity. Additionally, we performed a subgroup analysis on the primary outcome according to the different perioperative management protocols (fast-track vs. standard care).

## 3. Results

### 3.1. Study Selection and Characteristics

**Figure 1:**
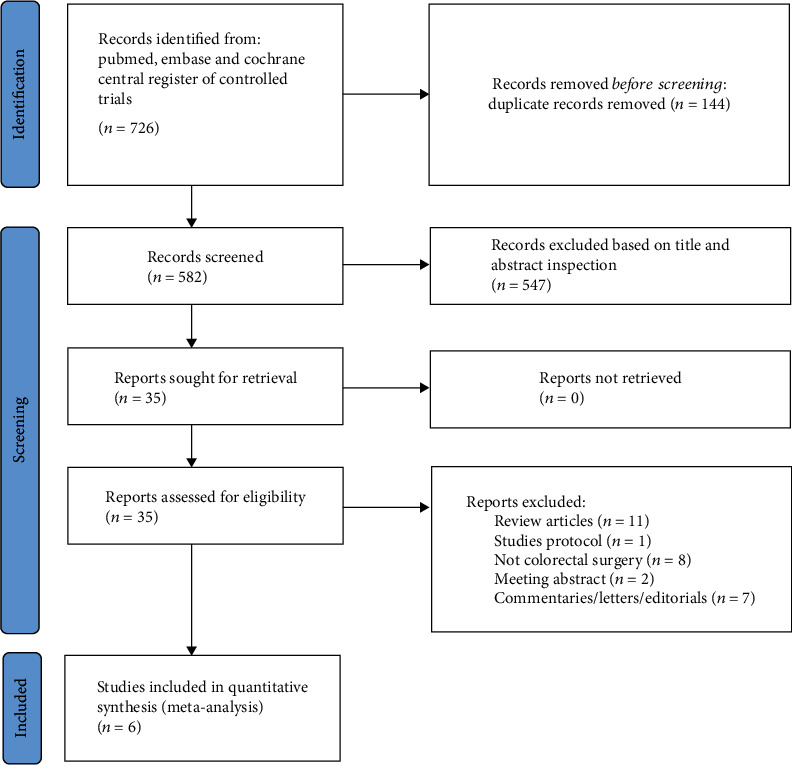
PRISMA flow diagram of the study search process.

A total of 726 studies were identified after an initial search. After the removal of duplicates and irrelevant studies, six RCTs capturing 416 patients who underwent colorectal surgery (207 patients with the administration of coffee/caffeine; 209 patients with the administration of water or tea) were included for further analysis [13, 14, 21–24]. The PRISMA flow diagram for the literature search is graphically represented in Figure 1. Characteristics of each trial are presented in Table 1. Most RCTs were single-center (*n* = 5/6 (83.3%)), including surgery for malignant or benign colorectal diseases (*n* = 4/6 (66.7%)), using a laparoscopic approach (*n* = 4/6 (66.7%)). Three studies applied the principles of fast-track surgery in perioperative management [13, 14, 24]. The patients were fed nutritious fluids within 48 h after surgery in five studies [13, 14, 22–24]. One study reported the administration of nutritious fluids on the third postoperative day [21]. The postoperative mobilization schedule was standardized across the studies. Caffeinated, decaffeinated coffee, or caffeine citrate was administered three times a day after surgery. The patients in the control group received water or a tee. In five studies [13, 14, 22–24], the intervention beverage was administered in the morning of the first postoperative day, while in one study [21], it was administered on the second postoperative day when the nasogastric tube was removed.

### 3.2. Quality Assessment

**Figure 2:**
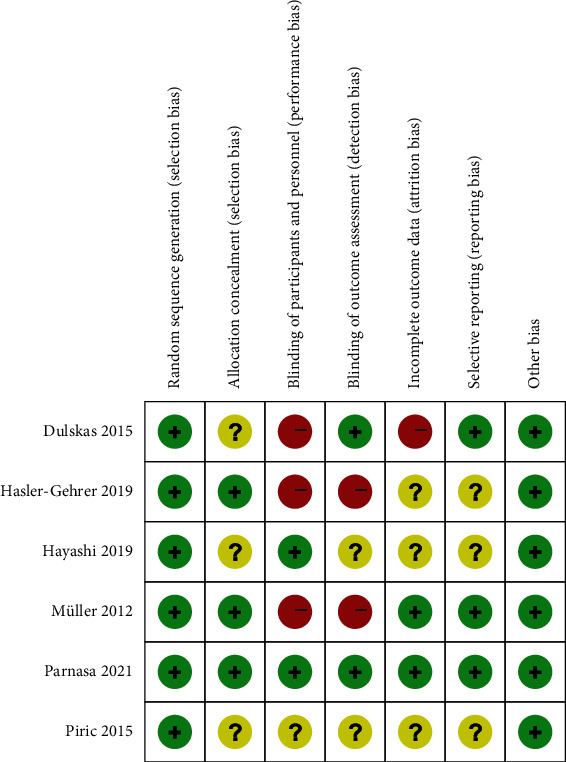
Risk of bias summary. Author's judgement about risk of bias for each included study, presented as high (+), low (-), or unclear (?).

The risk of bias was evaluated as low to moderate in six trials (Figure 2). Most trials had the same limitations, mainly focused on blinding of patients and evaluators because coffee has a unique aroma and color when compared with water. Only one study was double-blinded because the ampoules of caffeine citrate solution were applied in the trial [23].

### 3.3. Primary Outcomes

**Figure 3:**
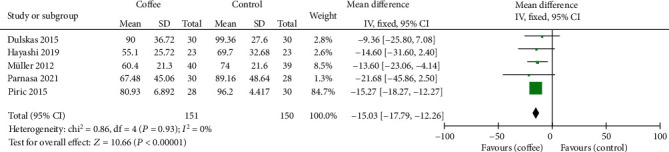
Forest plot of the time to first defecation.

The time to first defecation was available in all the included studies. Coffee shortened the time to first defecation with a *P* value of 0.008 in Hasler-Gehrer's study, but the outcome was reported as median and 95% CIs, which could not be transformed to mean and standard deviation. The meta-analysis of the other five trials revealed that the administration of coffee shortened the time to first defecation (MD = −15.03 h; 95% CI: -17.79, -12.26; *P* < 0.00001), with no heterogeneity (*I*^2^ = 0%) (Figure 3).

### 3.4. Secondary Outcomes

**Figure 4:**
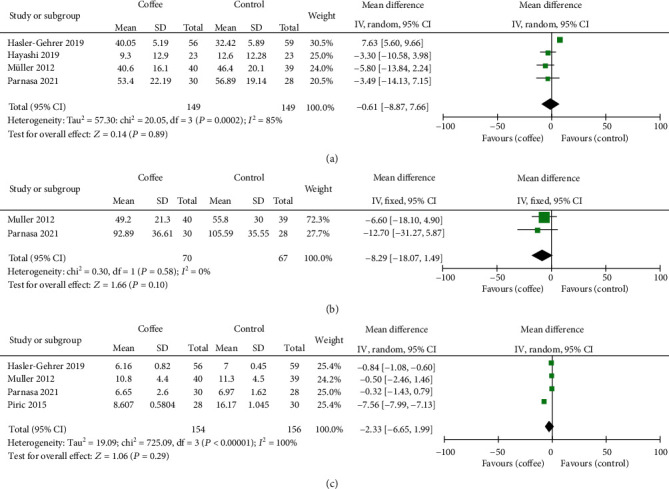
Forrest plot of (a) time to first flatus, (b) time to tolerance of solid food, and (c) length of hospital stay.

The time to first flatus was reported in four trials (*n* = 298). The pooled result provided that there were no differences between the two compared groups (MD = −0.61 h; 95% CI: -8.87, 7.66; *P* = 0.89), with remarkable heterogeneity (*I*^2^ = 85%) (Figure 4).

The time to tolerance for solid food was reported in two trials (*n* = 137). The pooled result provided that there were no differences between the two groups (MD = −8.92 h, 95% CI: -18.07, 1.49; *P* = 0.10), with no heterogeneity (*I*^2^ = 0%) (Figure 4).

Length of hospital stay was reported in four trials (*n* = 310). The pooled result provided that there were no differences between the two groups (MD = −2.33 h, 95% CI: -6.65, 1.99; *P* = 0.29), with remarkable heterogeneity (*I*^2^ = 100%) (Figure 4).

The use of laxatives for gastrointestinal motility was reported in three trials (*n* = 252). The pooled result provided that there were no differences between the two groups (RR = 0.76; 95% CI: 0.56, 1.04; *P* = 0.08), with low heterogeneity (*I*^2^ = 28%) (Table 2).

The nasogastric tube reinsertion during the postoperative period was reported in five trials (*n* = 370). The pooled result provided that there were no differences between the two groups (RR = 0.87; 95% CI: 0.44, 1.72; *P* = 0.68), with no heterogeneity (*I*^2^ = 0%) (Table 2).

The need for reoperation was reported in three trials (*n* = 195). The pooled result provided that there were no differences between the two groups (RR = 0.40; 95% CI: 0.08, 1.99; *P* = 0.26), with no heterogeneity (*I*^2^ = 0%) (Table 2).

Postoperative complications were reported in five trials (*n* = 358). The pooled result provided that there were no differences between the two groups (RR = 0.87; 95% CI: 0.58, 1.31; *P* = 0.52), with low heterogeneity (*I*^2^ = 11%) (Table 2).

The presence of an anastomotic fistula was reported in two trials (*n* = 194). The pooled result provided that there were no differences between the two groups (RR = 0.43; 95% CI: 0.12, 1.62; *P* = 0.21), with low heterogeneity (*I*^2^ = 27%) (Table 2).

### 3.5. Additional Analysis

Sensitivity analysis demonstrated that the primary outcome has no essential changes after excluding one study at a time. The heterogeneity in the time to first flatus was eliminated after excluding the study by Hasler-Gehrer, but the difference between the two groups remained statistically insignificant (MD = −4.23 h; 95% CI: -9.05, 0.58; *P* = 0.08; *I*^2^ = 0%). Heterogeneity in the use of laxatives and LOS was eliminated after excluding the study by Piric, and coffee consumption was associated with lower need of laxatives (RR = 0.66; 95% CI: 0.45, 0.95; *P* = 0.03; *I*^2^ = 0%) and shorter LOS (MD = −0.81 h; 95% CI: -1.05, -0.58; *P* < 0.00001; *I*^2^ = 0%).

**Figure 5:**
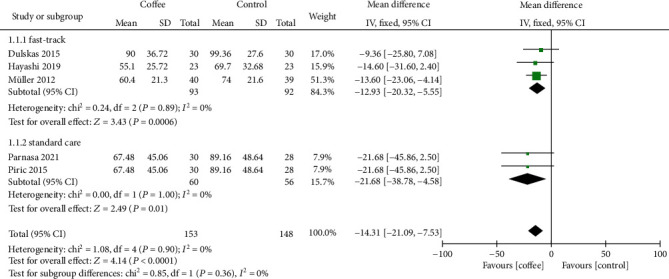
Subgroup analysis of the time to first defecation by type of perioperative management protocol.

In the subgroup analysis, the time to first defecation was not significantly different (*P* = 0.36) between patients treated by the fast-tract protocol and standard care protocol for perioperative management (Figure 5).

## 4. Discussion

POI is a major clinical and economic complication of colorectal surgery. Although POI is usually self-resolving, considerable efforts have been made to minimize the duration of POI due to its negative impact on patients. However, optimal treatments to prevent POI remain limited. This meta-analysis demonstrated a small effect of coffee in shortening POI after colorectal surgery. The pooled results revealed that postoperative coffee consumption shortened the time to first defecation. Additionally, there was no statistically significant difference in the time to first flatus, time to tolerance for solid food, LOS, use of laxatives, and reinsertion of nasogastric tube between the two groups. In the included trials, there were no adverse events associated with coffee. Meanwhile, there was no statistical difference in the need for reoperation, postoperative complications, and anastomotic leak between the two groups. Given the normal amount of coffee consumed and its safety, there should be no great concern.

The colonic motor activity is accelerated in 4 min after coffee consumption, while drinking water has no similar effect [12]. Rao et al. found that caffeinated coffee stimulates colon motility to an extent comparable to that of high-calorie foods [25]. Although the physiologic effects of coffee have been extensively studied, information on its effect on the bowel is not fully understood. While the most likely stimulant is caffeine, decaffeinated coffee stimulates bowel peristalsis in a previous study [25]. Parnasa et al. reported that caffeine significantly reduced the time to the first postoperative bowel movement when other chemical components were excluded [23]. Several mechanisms have been proposed: caffeine promotes postoperative gastrointestinal recovery through vasodilation [26, 27], improvement of POI by vagus nerve stimulation [28, 29], promoting the release of gastrin, which may cause the need for defecation shortly after ingestion [30]. Researchers believe that this laxative effect is caused by not only caffeine. Dulskas et al. showed that decaffeinated coffee was more effective in shortening the time to first bowel movement compared to caffeinated coffee, suggesting that a new active ingredient may have been formed during decaffeination [13]. Some theories suggest that the stimulant effect on the colon may be caused by other active substances, such as chlorogenic acid [31]. Chlorogenic acid can inhibit the formation of edema, leading to pain and improvement of pain after inflammatory reactions through anti-inflammatory effects [32, 33]. Piric et al. reported that C-reactive protein was significantly lower in the coffee-consuming group than in the control group on the third postoperative day, and there was a positive correlation between CRP level and the time to first defecation [21]. The abovementioned effects of coffee combined with effective postoperative analgesia can be beneficial to patient mobilization; thus, reducing the possibility of postoperative intestinal paralysis.

Our study found that coffee consumption only shortened the time to first defecation but had no effect on some other outcomes. First, although some of the results were not statistically significant, the results suggested that coffee consumption was more likely to reduce the need for laxatives and time to tolerance for solid food. One important reason for this result is that the vast majority of patients underwent laparoscopic surgery. Laparoscopic colorectal surgery has been extensively studied and has consistently improved many outcomes compared to laparotomy in recent years [34]. The use of drugs to control postoperative pain, as well as prolonged visceral manipulation and environmental exposure, resulted in longer POI after open surgery compared to laparoscopic colorectal surgery [35, 36]. Second, postoperative management in the included studies followed the principles of fast-track surgery. Multimodal fast track rehabilitation has been widely used in colorectal surgery, and all these measures have shown good results, such as reduced hospital stays and improved patient comfort [37, 38]. There was no difference in the time to first defecation between the patients treated by the fast-track protocol and those treated by standard care in the subgroup analysis. However, due to the small number of studies included, caution is necessary while interpreting the results. Third, whether the amount of coffee is sufficient enough to have an effect. In previous studies, patients consumed 240-280 ml of coffee at a time [25, 39]. Fourth, some uncontrolled and unmeasurable confounding factors might produce heterogeneity among studies, such as postoperative ambulation and opioid treatment.

The sensitivity analysis revealed that the heterogeneity was introduced by the inclusion of studies by Hasler-Gehrer and Piric alone. For example, tea was considered a control substance in both of these studies. The investigators had hypothesized that tea would accelerate the gastrointestinal transit through some ingredients, such as theophylline and thearubigin [40, 41]. To make the two groups comparable, caffeine-containing tea was excluded in Hasler-Gehrer's study. Importantly, Hasler-Gehrer reported a relatively high proportion of violation of coffee consumption in the control group. Meanwhile, Piric reported that coffee or water was provided on the second postoperative day after the nasogastric tube was removed and a fluid diet was started on the third postoperative day, which would delay the patient's food intake considerably. Moreover, some investigators reported that avoiding nasogastric tubes and early oral feeding can lead to earlier recovery of bowel function [42, 43]. Lastly, Piric also disclosed that rates of manual anastomosis and right hemicolectomy were significantly higher in the control group. These factors may have influenced the observed results.

There are some barriers to the implementation in practice of the studies, such as compliance with the study protocol and coffee standardization. Although patients with expected compliance deficits were excluded during the screening phase of the study, there was still a lack of compliance, such as off-protocol coffee consumption in the control group and refusion of coffee consumption in the coffee group. Hasler-Gehrer et al. reported more compliance by patients in the control group compared with coffee group at two time-points, which may indicate a stronger effect of coffee [22]. Coffee preparation also varied between studies, including the use of coffee capsules, instant coffee, and caffeine citrate. To reduce heterogeneity, our study included caffeinated coffee-consuming patients in the study by Dulskas. Likewise, the time to first defecation was stable after the study by Parnasa was excluded (MD = −14.94 h; 95% CI: -17.72, -12.16; *P* < 0.00001; *I*^2^ = 0%), in which caffeine citrate was used as an intervention substance. Future studies need to standardize coffee preparation to better evaluate the reliability of the results.

A clear definition of POI is currently lacking [44]; most included studies reported the time to first bowel movement as the time from the end of the operation to the first bowel movement in the present review, which may be affected by unblinded outcome assessors. In a study by Hayashi et al., patients took a radioopaque marker capsule orally in the morning of surgery, and radiographs were obtained daily [24]. The average number of evacuated markers 26 h after administration and the average number of markers that passed through the small intestine 6 h after administration were significantly higher after coffee consumption compared with water. Therefore, more objective methods are needed to evaluate the efficacy of coffee consumption on the recovery of gastrointestinal function after surgery.

Our review has some limitations. First of all, the sample size was small crossed the trials. Future larger multicenter studies are needed to conduct subgroup analyses of different patient characteristics and surgical approaches. Second, the dose-response relationship was unable to assess between the coffee dosage and outcome, and the standardized coffee is needed to determine the optimal dosage of coffee. Third, we could not perform a subgroup analysis of caffeinated and decaffeinated coffee. More studies are needed to investigate the chemical components of coffee that are beneficial for improving gastrointestinal function. Fourth, there was considerable heterogeneity between different studies. For instance, factors including perioperative management, control substance, and coffee preparation methods varied among studies. Future studies need more harmonization to the possible extent to assess the effect of coffee more accurately. Finally, our results may not be applicable to all people, as the chemical compounds in coffee may vary according to region, type of bean, brewing, and roasting method.

## 5. Conclusion

Coffee consumption after elective colorectal surgery is inexpensive and safe. Although the mechanism of action of coffee is not fully understood, the available studies suggest that coffee consumption is associated with shortened time to first defecation and may reflect a shorter recovery time of bowel motility. Nevertheless, a large-sample, multicenter, tightly controlled randomized clinical trial is needed to offer a more accurate evaluation of the efficacy of coffee in patients undergoing elective colorectal surgery.

## Figures and Tables

**Table 1:** Characteristics of included studies.

Author	Year	Center	Location	Participants			Surgical information				Intervention				
				Sample size (I/C)	Sex (M:F I/C)	Age (I/C, year)	Fast-track surgery	Colorectal pathology (M:B I/C)	Type of surgery	Operation time (range) (I/C, min)	Coffee	Volume (ml)	Drink time (min)	Frequency	Control
Muller	2012	Multicenter	Germany	40/39	25 : 15/19 : 20	65/59	Applied	23 : 17/22 : 17	Open and laparoscopy	173 ± 56/183 ± 57	Caffeinated	100	10	TDS	Water
Dulskas	2015	Single-center	Lithuania	30/(DC:30/W:30)	16 : 14/16 : 14/16 : 14	67.3/62.4/66.3	Applied	30 : 0/30 : 0/30 : 0	Laparoscopy	102.0 ± 37.2/103.0 ± 42.5/98.0 ± 35.2	Caffeinated DC	100	10	TDS	Water
Piric	2015	Single-center	Bosnia and Herzegovina	28/30	17 : 11/17 : 13	63.57/62.67	NM	25 : 3/28 : 2	Open	139.3 ± 6.764/130.8 ± 6.798	Caffeinated	100	10	TDS	Tea
Hasler-Gehrer	2019	Single-center	Switzerland	56/59	31 : 25/28 : 31	63/69	NM	23 : 33/29 : 30	Laparoscopy	160 (136–185) /150 (130–180)	Caffeinated	150	NM	TDS	Tea
Hayashi	2019	Single-center	Japan	23/23	5 : 18/7 : 16	74.0/80.2	Applied	0 : 23/0 : 23	Laparoscopy	181.6 (120-266)/177.0 (107-333)	Caffeinated	100	10	TDS	Water
Parnasa	2021	Single-center	Israel	30/28	15 : 15/14 : 14	56.90/55.36	NM	NM	Laparoscopy	NM	Caffeine citrate	50^∗^	NM	TDS	Apple-flavored water

^∗^100 mg of caffeine citrate was diluted in 50 ml of apple-flavored water. I: coffee group; C: control group; sex (M: male; F: female); colorectal pathology (M: malignancy; B: benign); DC: decaffeinated coffee; W: water; NM: not mentioned; TDS: three times per day.

**Table 2:** Meta-analysis of dichotomous secondary outcomes.

Outcome	No. of trials	No. of patients		No. of events		RR (95% CI)	*I* ^2^ (%)	*P*
		Coffee	Control	Coffee	Control			
Use of laxatives	3^14, 21, 22^	124	128	42	57	0.76 (0.56, 1.04)	28	0.08
Reinsertion of nasogastric tube	5^13, 14, 22-24^	184	186	13	15	0.87 (0.44, 1.72)	0	0.68
Need of reoperation	3^14, 21, 23^	98	97	2	5	0.40 (0.08, 1.99)	0	0.26
Postoperative complications	5^13, 14, 21, 22, 24^	177	181	34	40	0.87 (0.58, 1.31)	11	0.52
Anastomotic fistula	2^14, 22^	96	98	3	7	0.43 (0.12, 1.62)	27	0.21

Meta-analysis of dichotomous secondary outcomes among trials with risk ratios (RR), 95% confidence intervals (CI), and heterogeneity (*I*^2^).

## Data Availability

The data supporting this meta-analysis are from previously reported studies and datasets, which have been cited. The processed data are available from the corresponding author upon request.
